# Spectral clustering using Nyström approximation for the accurate identification of cancer molecular subtypes

**DOI:** 10.1038/s41598-017-05275-3

**Published:** 2017-07-07

**Authors:** Mingguang Shi, Guofu Xu

**Affiliations:** School of Electric Engineering and Automation, Hefei University of Technology, Hefei, Anhui 230009 China

## Abstract

A major challenge in clinical cancer research is the identification of accurate molecular subtype. While unsupervised clustering methods have been applied for class discovery, this clustering method remains a bottleneck in developing accurate method for molecular subtype discovery. In this analysis, we hypothesize that spectral clustering method could identify molecular subtypes in correlation with survival outcomes. We propose an accurate subtype identification method, Cancer Subtype Identification with Spectral Clustering using Nyström approximation (CSISCN), for the discovery of molecular subtypes, based on spectral clustering method. CSISCN could be used to improve gene expression-based identification of breast cancer molecular subtypes. We demonstrated that CSISCN identified the molecular subtypes with distinct clinical outcomes and was valid for the number of molecular subtypes. Furthermore, CSISCN identified molecular subtypes for improving clinical and molecular relevance which significantly outperformed consensus clustering and spectral clustering methods. To test the general applicability of the CSISCN, we further applied it on human CRC datasets and AML datasets and demonstrated superior performance as compared to consensus clustering method. In summary, CSISCN demonstrated the great potential in gene expression-based subtype identification.

## Introduction

Identifying the subtype of cancer is one of the leading area of study in clinical cancer research. The use of accurate subtype identification typically helps to determine the appropriate therapy and thus improves survival rate for cancer patients. To date, the rapid development of high-throughput platforms such as gene expression profiling^1, 2^, human whole-genome sequencing^3, 4^ and whole-exome sequencing^5^ have been applied to cancer data for the prioritization of expression-based signatures^6, 7^, the discovery of recurrent mutations^3, 4^, the identification of molecular subtypes^1, 8^, the development of prognosis model^9, 10^ and the selection of patients likely benefit from particular targeted therapies^11^. In particular, advances in cancer genomics studies have revealed the marked clinical and molecular heterogeneity with regard to responses from treatment and survival outcomes^12, 13^. However, the heterogeneity in tumor samples poses considerable challenges for the evaluation of prognosis and selection of an appropriate treatment for each individual patient^14^. Thus, there is urgent need to provide the accurate subtype identification method for developing the prognostic and therapeutic strategies.

Traditional unsupervised clustering methods have showed great potential in identifying modular network^15^, discovering molecularly distinct subtypes^16–18^ and identifying oncogenic pathway signatures^11^ in cancer research. Specifically, consensus clustering method has been widely used for class discovery^19, 20^ and the identification of consensus molecular subtypes^8, 21^. While traditional clustering algorithms are mainly founded on Euclidean geometry and unable to treat nonlinear structure in data, spectral clustering could adapt to geometries in a broader range due to the identification of non-convex patterns and linearly non-separable clusters^22^. Importantly, spectral clustering has been widely used in machine learning and pattern recognition^22–25^. It partitions the points into distinct clusters based on the eigenstructure of the similarity matrix. Accordingly, the points have high similarity in the same cluster and low similarity in different clusters^26^. Despite its good performance, spectral clustering is often limited in its application for large-scale problems due to its high computational complexity^27^. To address this challenge, the spectral clustering using Nyström approximation is presented to reduce the computational cost of the matrix decomposition and improve the clustering accuracy^28, 29^.

In this paper, we aimed to develop and evaluate spectral clustering method using Nyström approximation for identifying molecular subtypes of cancer. We investigated whether this method could identify molecular subtypes for improving clinical and molecular relevance. We proposed an accurate subtype identification method, Cancer Subtype Identification with Spectral Clustering using Nyström approximation (CSISCN), for the discovery of molecular subtypes, based on spectral clustering method. We first started with the discovery for molecular subtypes of breast cancer patients based on gene expression profiles (GEPs). Then, we demonstrated that, 1) The CSISCN identified the molecular subtypes with distinct clinical outcomes; 2) The CSISCN was valid for the number of molecular subtypes; and 3) The CSISCN identified molecular subtypes for improving clinical and molecular relevance as compared to the consensus clustering and spectral clustering methods. To test the general applicability of the CSISCN, we further applied it on human CRC datasets and AML datasets and demonstrated superior performance as compared to consensus clustering and spectral clustering methods.

## Methods

### Gene expression datasets of cancer patients

Breast cancer consisted of distinct biological subtypes including HER2, ER and PR for different prognostic and therapeutic implications. We have collected breast cancer gene expression datasets using the Affymetrix U133A platforms from public resources. The gene expression datasets GSE25055^30^, GSE25065^30^ and GSE6532^31^ were downloaded from the Gene Expression Omnibus (GEO) database. Neoadjuvant study of 310 HER2-negative breast cancer cases in GSE25055 and 198 HER2-negative breast cancer cases in GSE25065 were treated with taxane-anthracycline chemotherapy pre-operatively and endocrine therapy. The clinically distinct molecular subtypes were identified in estrogen receptor positive breast carcinomas GSE6532. In this study, tumor samples from GSE25055 were used as training cohort, and those from two gene expression datasets GSE25065 and GSE6532 were used as independent validation cohorts.

Mutations in specific genes APC, KRAS, PIK3CA and TP53 allowed the identification of prognostic subgroups in colorectal cancer (CRC). The TCGA (The Cancer Genome Atlas) study recently reported three transcriptomic subtypes of CRC, which were designated as “microsatellite instability/CpG islandmethylator phenotype” (MSI/CIMP), “invasive”, and “chromosomal instability” (CIN)^32^. The training cohort GSE17536^33, 34^ including 111 samples in CRC patients was obtained from GEO database. In our study, we analyzed independent validation cohort GSE17537^33, 34^ downloaded from GEO database. Stage І and IV samples were excluded from this study. All these two CRC datasets were generated on the Affymetirx U133 plus 2.0 platform. The metastasis gene expression profiles GSE17536 (Moffitt patients) and GSE17537 (VMC patients) were developed from highly invasive mouse colon cancer cells and non-invasive colon cancer cells respectively.

Acute myeloid leukemia (AML) patients were classified into M0-M7 subgroups with FAB (French–American–British) criteria^35^. For AML, two gene expression datasets including GSE12417^36^ (HG-U133A) and GSE10358^37^ (HG-U133Plus2) were downloaded from GEO database. In GSE12417, 163 samples of bone marrow or peripheral blood mononuclear cells were developed from adult patients with untreated AML. The high-throughput sequencing using genomic DNA or RNA were created from the bone marrow (tumor) and matched skin biopsy samples (germline) from over 300 patients with de novo AML in GSE10358. GSE12417 was used as training cohort and GSE10358 was used as test cohort respectively.

For tumor gene expression datasets, all Affymetrix based CEL files were normalized using the Robust MultiChip Analysis (RMA) algorithm^38^ from the R Bioconductor package. Probe set identifiers (IDs) were mapped to gene symbols with the mapping from the GEO database. The probe set with the largest interquartile range (IQR) was selected owing to its high variation across samples, when multiple probe sets were mapped to the same gene. Probe sets were eliminated when they were mapped to multiple genes. The Z-score transformation was used as a normalization procedure to standardize the expression values of each gene. The datasets were performed separately to ensure their independency. The clinical characteristics of tumor samples with breast cancer, CRC and AML are listed in Table 1.

**Table 1:** Microarray datasets for CSISCN development and validation.

Tissue	GEO	#Samples	Survival event	#Genes
Breast cancer	GSE25055	310	DRFS (66 1, 244 0)	12694
Breast cancer	GSE25065	198	DRFS (45 1, 153 0)	12694
Breast cancer	GSE6532	241	DRFS (82 1, 159 0)	12694
CRC	GSE17536	111	RFS (31 1, 80 0)	19468
CRC	GSE17537	55	RFS (19 1, 36 0)	19825
AML	GSE12417	163	OS (102 1, 61 0)	11796
AML	GSE10358	91	OS (45 1, 46 0)	12694

(DRFS: distant relapse free survival, RFS: relapse-free survival, OS: overall survival, 1: recurrence, 0: non-recurrence).

### Spectral clustering using Nyström approximation

Input: data points ${\rm{X}}={\{x}_{1},\ldots ,{{\rm{x}}}_{{\rm{n}}}\}$ representing gene expression levels of patients; $\ell$: number of random samples; σ: Gaussian function scaling parameter; k: number of identified clusters; n: the number of patients; ${\rm{k}} < \ell  < {\rm{n}}$, 1 ≤ i, j ≤ n.

1. Form the similarity matrix ${\rm{S}}\in {{\rm{R}}}^{{\rm{n}}\times {\rm{n}}}$ defined by ${{\rm{s}}}_{{\rm{ij}}}=\exp (-\parallel {{\rm{x}}}_{{\rm{i}}}-{{\rm{x}}}_{{\rm{j}}}{\parallel }^{2}/2{{\rm{\sigma }}}^{2})$ if ${\rm{i}}\ne {\rm{j}}$, and s_ii_ = 0.
2. Let A represent the $\ell \times \ell$ matrix of similarities between the sample points, B represent the $\ell \times ({\rm{n}}-\ell )$ matrix of affinities between the $\ell$ sample points and the $({\rm{n}}-\ell )$ remaining points, and C represent the submatrix. The dense similarity matrix S_d_ is the reconstitution of the similarity matrix S and constructed with $${{\rm{S}}}_{{\rm{d}}}=[\begin{array}{cc}{\rm{A}} & {\rm{B}}\\ {{\rm{B}}}^{{\rm{T}}} & {\rm{C}}\end{array}]$$.
3. Assume $${\rm{W}}=[\begin{array}{c}{\rm{A}}\\ {{\rm{B}}}^{{\rm{T}}}\end{array}]$$, and define $$\tilde{{\rm{S}}}\approx {{\rm{S}}}_{{\rm{d}}}={{\rm{WA}}}^{-1}{{\rm{W}}}^{{\rm{T}}}=[\begin{array}{cc}{\rm{A}} & {\rm{B}}\\ {{\rm{B}}}^{{\rm{T}}} & {{\rm{B}}}^{{\rm{T}}}{{\rm{A}}}^{-{\rm{1}}}{\rm{B}}\end{array}]$$ with Nyström approximation. W represents the ${\rm{n}}\times \ell$ matrix consisting of A and B^T^.
4. Calculate the diagonal matrix $$\tilde{{\rm{D}}}={\rm{diag}}\,([\begin{array}{c}{{\rm{A1}}}_{\ell }+{{\rm{B1}}}_{{\rm{n}}-\ell }\\ {{\rm{B}}}^{{\rm{T}}}{{\rm{1}}}_{\ell }+{{\rm{B}}}^{{\rm{T}}}{{\rm{A}}}^{-{\rm{1}}}{{\rm{B1}}}_{{\rm{n}}-\ell }\end{array}])$$.
5. Define Laplacian matrix $\tilde{{\rm{L}}}={\rm{I}}-{\tilde{{\rm{D}}}}^{-{\rm{1}}/{\rm{2}}}{\tilde{{\rm{S}}}\tilde{{\rm{D}}}}^{-{\rm{1}}/{\rm{2}}}$.
6. Define ${\rm{R}}=\bar{{\rm{A}}}+{\bar{{\rm{A}}}}^{-\frac{1}{2}}{\bar{{\rm{B}}}\bar{{\rm{B}}}}^{{\rm{T}}}{\bar{{\rm{A}}}}^{-\frac{1}{2}}$, where $\bar{{\rm{A}}}={\tilde{{\rm{D}}}}_{1:\ell ,1:\ell }^{-1/2}{A\tilde{{\rm{D}}}}_{1:\ell ,1:\ell }^{-1/2}$ and $\bar{{\rm{B}}}={\tilde{{\rm{D}}}}_{{\rm{1}}:\ell ,{\rm{1}}:\ell }^{-{\rm{1}}/{\rm{2}}}{B\tilde{{\rm{D}}}}_{\ell +{\rm{1}}:{\rm{n}},\ell +{\rm{1}}:{\rm{n}}}^{-{\rm{1}}/{\rm{2}}}$.
7. Calculate eigendecomposition of R, ${\rm{R}}={{\rm{U}}}_{{\rm{R}}}{{\rm{\Lambda }}}_{{\rm{R}}}{{\rm{U}}}_{{\rm{R}}}^{{\rm{T}}}$, ${{\rm{\Lambda }}}_{{\rm{R}}}$ is the eigenvalues with decreasing order and U_R_ is the eigenvectors.
8. Calculate $$\tilde{{\rm{V}}}=[\begin{array}{c}\bar{{\rm{A}}}\\ {\bar{{\rm{B}}}}^{{\rm{T}}}\end{array}]{\bar{{\rm{A}}}}^{-\frac{1}{2}}{({{\rm{U}}}_{{\rm{R}}})}_{:,1:{\rm{k}}}{({{\rm{\Lambda }}}_{{\rm{R}}}^{-\frac{1}{2}})}_{1:{\rm{k}},1:{\rm{k}}}$$ with the first k eigenvectors.
9. Define the normalized matrix $\tilde{{\rm{U}}}$ with ${\tilde{{\rm{u}}}}_{{\rm{il}}}=\frac{{\tilde{{\rm{V}}}}_{{\rm{il}}}}{\sqrt{{\sum }_{{\rm{r}}={\rm{1}}}^{{\rm{k}}}\,{\tilde{{\rm{V}}}}_{{\rm{ir}}}^{{\rm{2}}}}}$, where ${\rm{l}}=1,\ldots ,{\rm{k}}$.
10. Perform the k-means algorithm to cluster n rows of $\tilde{{\rm{U}}}$ into k groups. K-means algorithm minimize the objective function ${\sum }_{{\rm{i}}=1}^{{\rm{k}}}{\sum }_{{{\rm{u}}}_{{\rm{j}}}\in {{\rm{C}}}_{{\rm{i}}}}\parallel {{\rm{u}}}_{{\rm{j}}}-{{\rm{c}}}_{{\rm{i}}}{\parallel }^{2}$, where u_j_ is vectors corresponding to n rows of $\tilde{{\rm{U}}}$ and c_i_ is the centroid of all the points u_j_ belonging to cluster c_i_. We define ${{\rm{c}}}_{{\rm{i}}}=\frac{1}{|{{\rm{s}}}_{{\rm{i}}}|}{\sum }_{{{\rm{u}}}_{{\rm{j}}}\in {{\rm{s}}}_{{\rm{i}}}}{{\rm{u}}}_{{\rm{j}}}$, where ${{\rm{s}}}_{{\rm{i}}}=\{{{\rm{u}}}_{{\rm{p}}}:\parallel {{\rm{u}}}_{{\rm{p}}}-{{\rm{c}}}_{{\rm{i}}}{\parallel }^{2}\le \parallel {{\rm{u}}}_{{\rm{p}}}-{{\rm{c}}}_{{\rm{j}}}{\parallel }^{2}\}$.
11. K-means iterations terminated with the relative difference between the two values of the objective function less than 0.001.

### Subtype identification of CSISCN and consensus clustering approach

We proposed an accurate subtype identification method, Cancer Subtype Identification with Spectral Clustering using Nyström approximation (CSISCN), for the discovery of molecular subtypes, based on spectral clustering method. For spectral clustering using Nyström approximation, a Matlab implementation was used for this study. For tumor samples of training cohort, we set the parameter σ vary among the candidate set {20, 30, 40, 50} for each cancer type. Full gene symbols were used as outcome-related genes for input features. In the implementation of spectral clustering using Nyström approximation, we let the half sample size of training cohort as the number of random samples for each cancer type. The k-means algorithm was performed to identify the k clusters. The identified k clusters and real prognosis of the patients was assessed by the Kaplan-Meier survival curves and log-rank test. Each choice from the parameter σ was evaluated with log-rank p-value over 10 runs, and the parameter σ with smallest p-value was identified. The identified parameter σ was then performed to test on the independent validation dataset and the performance was evaluated with the Kaplan-Meier estimated survival curves.

For reference, we compared performance from the CSISCN approach to that from the state of the art unsupervised clustering method consensus clustering approach^19^. Consensus clustering has proved to be effective in solving different biological problems including gene expression-based class discovery^19^, identification of biologically functional modules in Protein–Protein Interaction (PPI) networks^39^, and cancer subtype discovery^40^. An R implementation of the ConsensusClusterPlus^41^ available in the ConsensusClusterPlus package was used for consensus clustering method. The pearson correlation coefficient distance was used with hierarchical clustering. The consensus clusters were identified as cancer subtypes from 100 resampling iterations of the hierarchical clustering, by using the full gene symbols (100%) and randomly selecting a fraction of the 80% samples. The identified cancer subtype and real prognosis of the patients was then assessed with survival analysis by the Kaplan-Meier survival curves and log-rank test. The number of consensus clusters was selected from k = 2 to k = 10 respectively.

### Survival analysis

The association between the molecular subtype and real prognosis of the patients was evaluated by the Kaplan-Meier survival curves and log-rank test. Standard Kaplan–Meier survival curves were generated for each cancer subtype, and the survival difference between molecular subtypes was statistically evaluated using the log-rank test. An R implementation in the survival package was used for survival analysis. P-values of less than 0.05 were considered statistically significant.

## Results

### Overview of the CSISCN development and evaluation workflow

**Figure 1:**
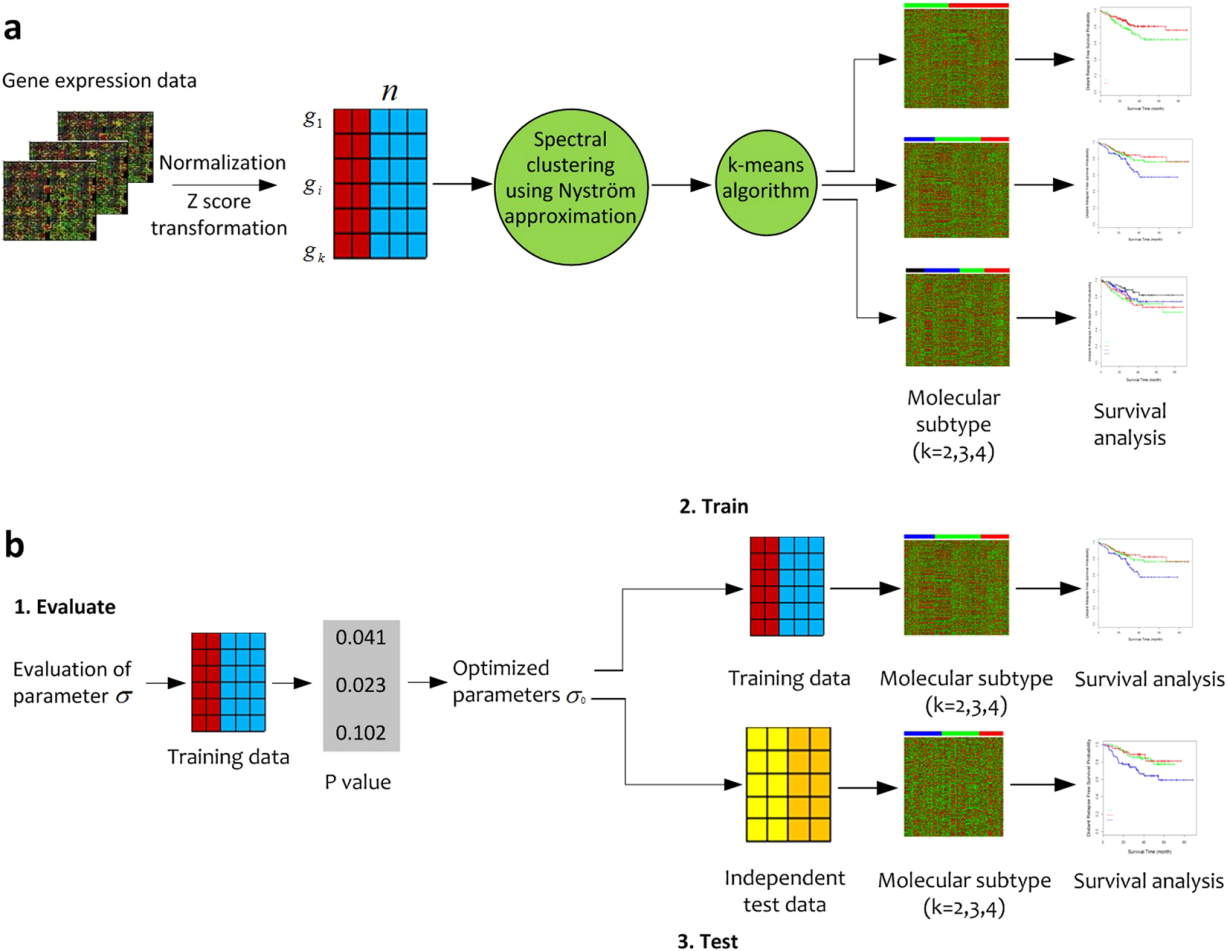
Workflow for the development and evaluation of the CSISCN. (**a**) Gene expression data are normalized and z-score transformed from breast cancer, CRC and AML. The CSISCN was developed with spectral clustering using Nyström approximation and k-means algorithm to identify molecular subtypes. (**b**) The evaluation of parameter vary among the candidate set, the clustering method is developed based on the optimal parameter and the testing procedure is then specified.

Figure 1 illustrates the overview of the CSISCN development and evaluation workflow. Microarray gene expression data on a specific cancer type were collected, normalized, and then z-score transformed separately. Molecular subtype of cancer was discovered from spectral clustering using Nyström approximation and k-means algorithm with the full gene symbols of GEPs. On the training set, we let the Gaussian function scaling parameter σ vary among the candidate set to construct the similarity matrix. CSISCN discovered the k clusters as molecular subtypes of cancer based on the identified optimal parameter. The association between identified molecular subtype and real prognosis of the patients was assessed by the Kaplan-Meier survival analysis. For CSISCN, the identified optimal parameter σ was then performed to test on the independent validation dataset. The k clusters were recognized as molecular subtypes to stratify the validation cohort and the prediction performance was then evaluated with the Kaplan-Meier survival curves and log-rank test. For tumor samples of validation dataset in each cancer type, we set the number of random samples with the half sample size of test cohort.

### The CSISCN identifies the molecular subtypes with distinct clinical outcomes

We presented the CSISCN to identify the molecular subtypes from tumor GEPs. We investigated whether CSISCN could identify the molecular subtypes in breast cancer as an example. GSE25055 was used as training cohort for clustering development. GSE25065 and GSE6532 were then used as two independent validation cohorts to validate the approach. For each parameter σ, log-rank p-values were generated with repeated ten times runs in order to obtain robust performance evaluation results. In this analysis, the parameter σ = 20 was identified with smallest p-value from training cohort and then performed to test on the independent validation dataset.

**Figure 2:**
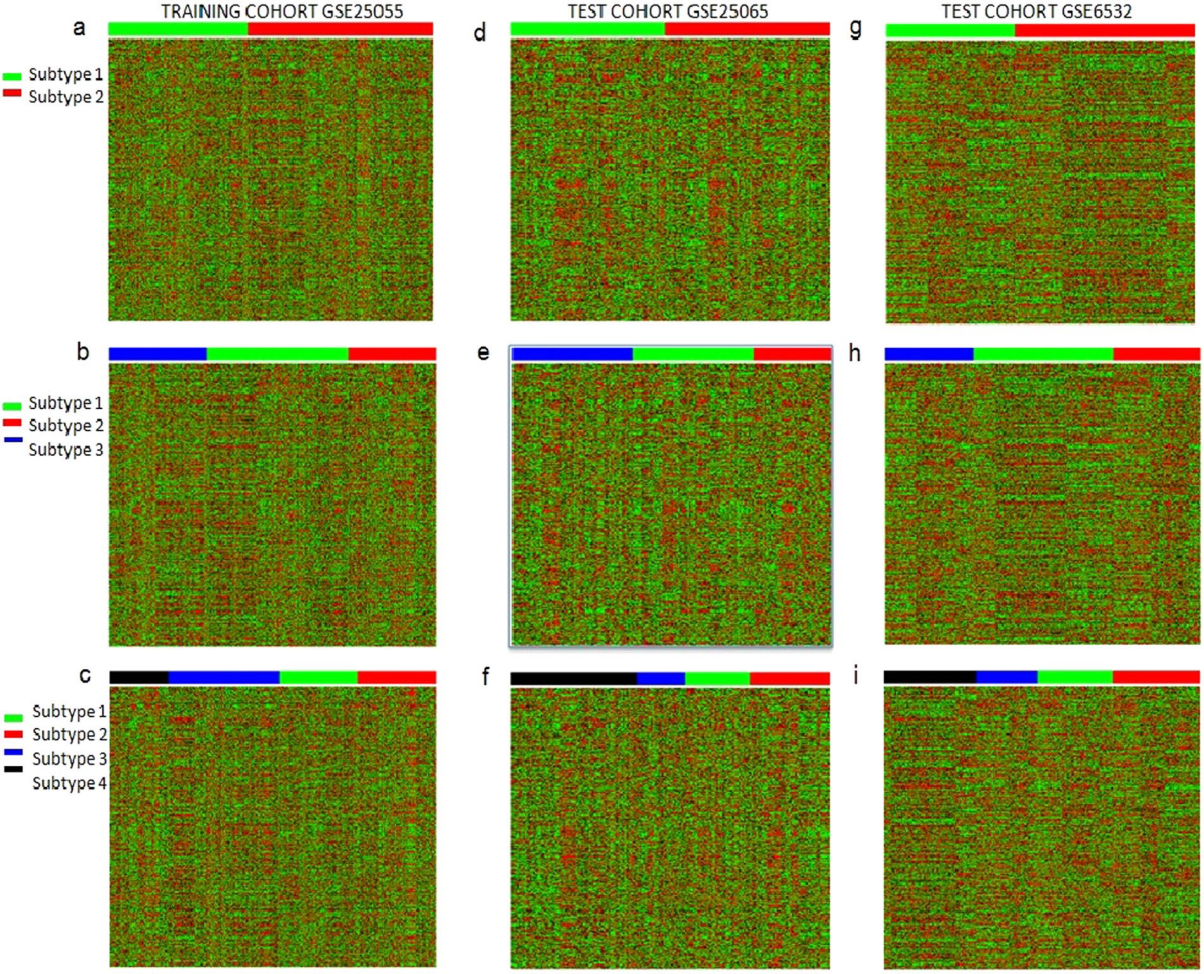
Heatmaps of subtype-discriminant gene expression profiles in the training dataset and in the independent test dataset. CSISCN identify k clusters as molecular subtypes of cancer. (**a**–**c**) Heatmaps are shown from two to four distinct subtypes in training dataset GSE25055. (**d**–**f**) Heatmaps are shown from two to four distinct subtypes in independent test dataset GSE25065. (**g**–**i**) Heatmaps are shown from two to four distinct subtypes in independent test dataset GSE6532.

To identify the difference in gene expression between molecular subtypes, we performed CSISCN to stratify the cancer patients into k clusters. Figure 2 showed the molecular subtypes with distinct cluster discriminating patterns of breast cancer. The heatmap further revealed the subtype based discriminative patterns of alterations in GEPs.

**Figure 3:**
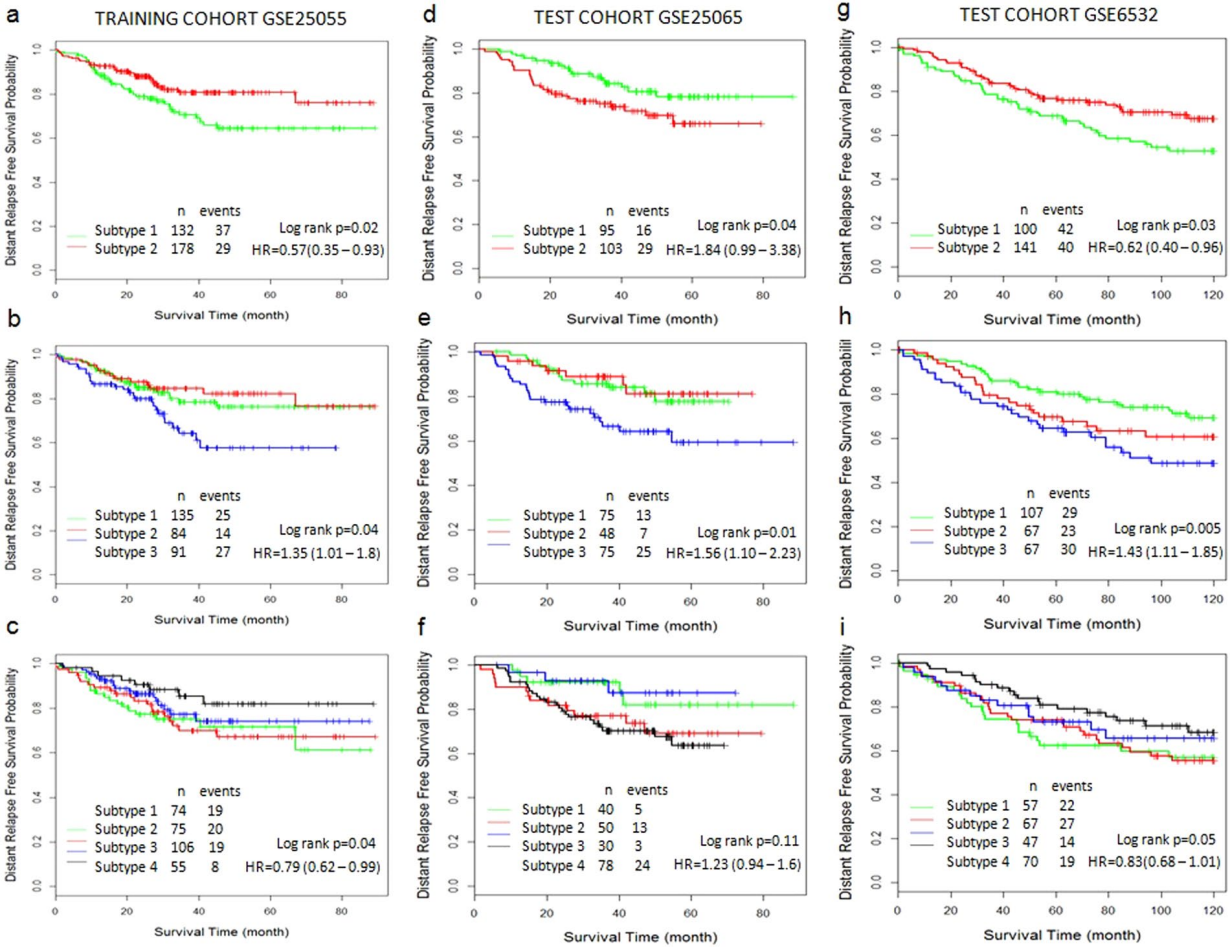
The molecular subtypes have distinct clinical outcomes in breast cancer. (**a**–**c**) Kaplan–Meier plot of distant relapse-free survival for two, three and four subtypes respectively in training dataset GSE25055. (**d**–**f**) Kaplan–Meier plot of distant relapse-free survival for two, three and four subtypes respectively in independent test dataset GSE25065. (**g**–**i**) Kaplan–Meier plot of distant relapse-free survival for two, three and four subtypes respectively in independent test dataset GSE6532. Hazard ratio (HR) was derived with 95% confidence interval.

**Figure 4:**
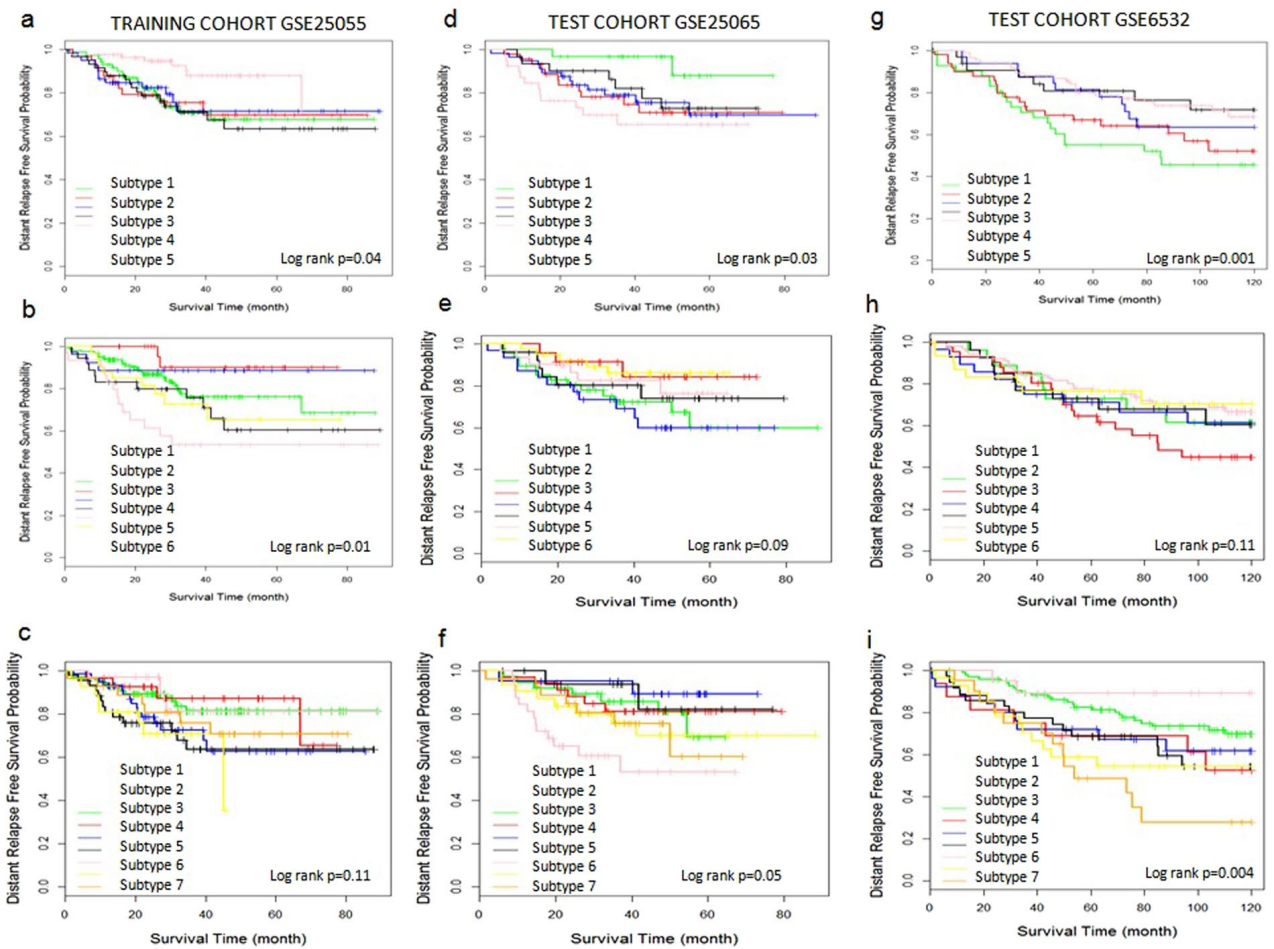
The molecular subtypes have distinct clinical outcomes in breast cancer. (**a**–**c**) Kaplan–Meier plot of distant relapse-free survival for five, six and seven subtypes respectively in training dataset GSE25055. (**d**–**f**) Kaplan–Meier plot of distant relapse-free survival from five subtypes to seven subtypes respectively in independent test dataset GSE25065. (**g**–**i**) Kaplan–Meier plot of distant relapse-free survival from five subtypes to seven subtypes respectively in independent test dataset GSE6532.

**Figure 5:**
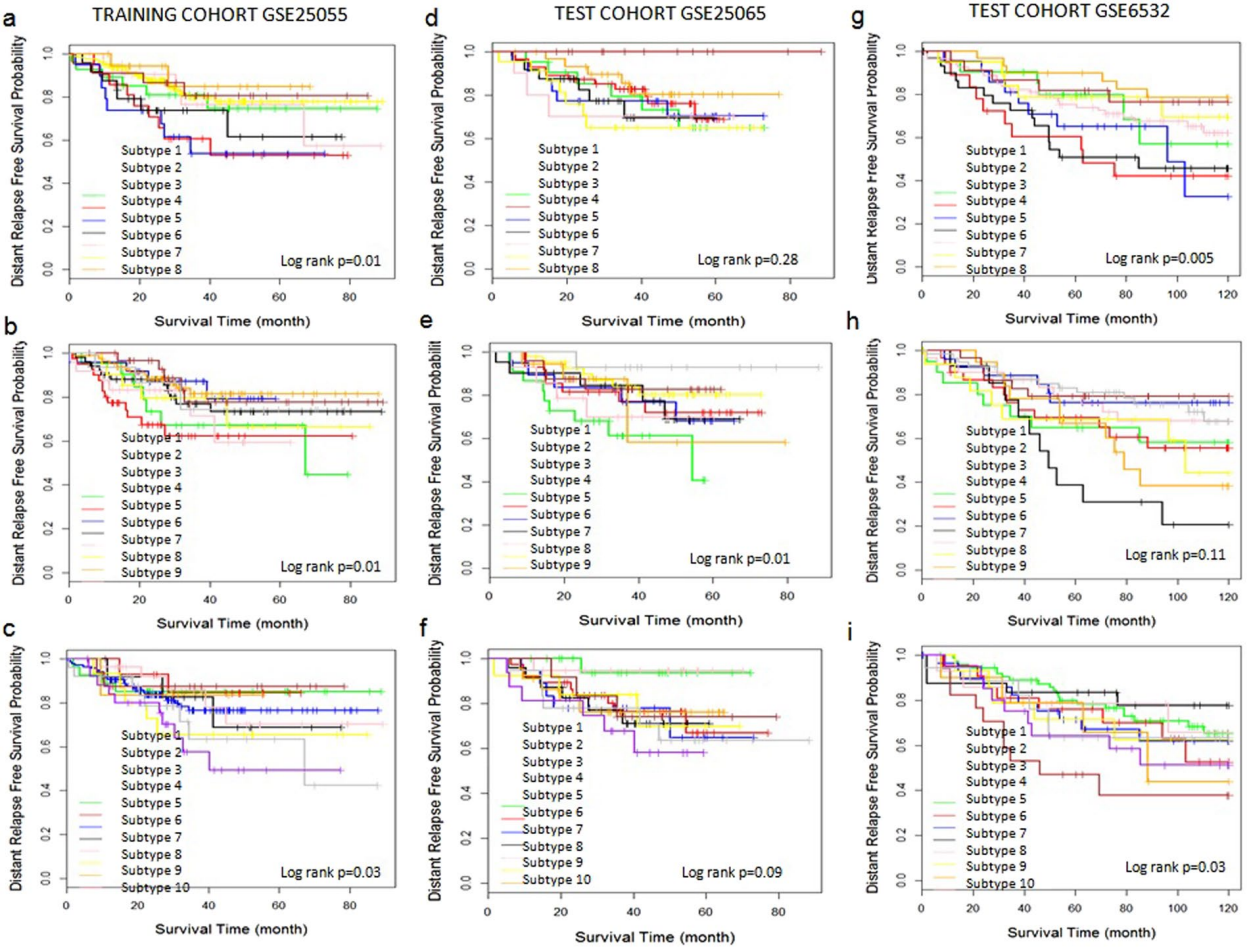
The molecular subtypes have distinct clinical outcomes in breast cancer. (**a**–**c**) Kaplan–Meier plot of distant relapse-free survival for eight, nine and ten subtypes respectively in training dataset GSE25055. (**d**–**f**) Kaplan–Meier plot of distant relapse-free survival from eight subtypes to ten subtypes respectively in independent test dataset GSE25065. (**g**–**i**) Kaplan–Meier plot of distant relapse-free survival from eight subtypes to ten subtypes respectively in independent test dataset GSE6532.

GSE25055 was used as training cohort to develop the CSISCN for identifying molecular subtypes. As shown in Fig. 3a, the subtype 1 group had significantly worse distant relapse-free survival than the subtype 2 group. The distant relapse free survival at 3 years was 70% for the subtype 1 group compared with 80% for the subtype 2 group. As shown in Fig. 3b, the patients were separated into three subtypes with significantly different distant relapse-free survival. The distant relapse free survival at 3 years was 78% for the subtype 1 group compared with 84% for the subtype 2 group and 64% for the subtype 3 group respectively. As shown in Fig. 3c, the patients were stratified into four subtypes with significantly different distant relapse-free survival. The distant relapse free survival at 3 years was 75% for the subtype 1 group as compared to 69% for the subtype 2 group, 77% for the subtype 3 group and 85% for the subtype 4 group respectively. To further test the generality of the method, we developed the CSISCN from GSE25055 for identifying from five to ten molecular subtypes. As shown in the Fig. 4, it illustrated that the patients were stratified into five molecular subtypes with significantly different relapse-free survival (Fig. 4a) and six molecular subtypes with significantly different relapse-free survival (Fig. 4b) respectively. Still, we observed that the patients were separated into eight molecular subtypes with significantly different distant relapse-free survival (Fig. 5a), nine molecular subtypes with significantly different distant relapse-free survival (Fig. 5b) and ten molecular subtypes with significantly different distant relapse-free survival (Fig. 5c) respectively.

Using optimized parameter σ based on training cohort, the CSISCN was developed to test on the independent dataset GSE25065. Figure 3d illustrated the subtype 2 group had significantly worse distant relapse-free survival than the subtype 1 group. The distant relapse free survival at 3 years was 84% for the subtype 1 group compared with 74% for the subtype 2 group. Figure 3e depicted the patients were separated into three subtypes with significantly different distant relapse-free survival. The distant relapse free survival at 3 years was 84% for the subtype 1 group compared with 85% for the subtype 2 group and 66% for the subtype 3 group respectively. We observed that the patients were separated into five molecular subtypes (Fig. 4d), seven molecular subtypes (Fig. 4f) and nine molecular subtypes (Fig. 5e) respectively with significantly different distant relapse-free survival when the CSISCN was applied for breast cancer gene expression datasets GSE25065.

To further validate the effect of the CSISCN, we developed the clustering method to test on the independent dataset GSE6532. We observed that the subtype 1 group had significantly worse distant relapse-free survival than the subtype 2 group (Fig. 3g). The distant relapse free survival at 3 years was 78% for the subtype 1 group compared with 83% for the subtype 2 group. Still, we noticed that the patients were separated into three subtypes with significantly different distant relapse-free survival (Fig. 3h). The distant relapse free survival at 3 years was 86% for the subtype 1 group compared with 79% for the subtype 2 group and 75% for the subtype 3 group respectively. Figure 3i showed that the patients were stratified into four subtypes with significantly different distant relapse-free survival. The distant relapse free survival at 3 years was 74% for the subtype 1 group as compared to 77% for the subtype 2 group, 81% for the subtype 3 group and 89% for the subtype 4 group respectively. As shown in the Figs 4 and 5, it illustrated that the patients were stratified into different molecular subtypes with significantly different relapse-free survival when the CSISCN was applied for breast cancer gene expression datasets GSE6532 (Figs 4g,i and 5g,i respectively).

Consequently, both training results and independent test results clearly demonstrated that the CSISCN was able to identify the molecular subtypes with significant differences in prognosis.

### The CSISCN is effective in CRC datasets and AML datasets

**Figure 6:**
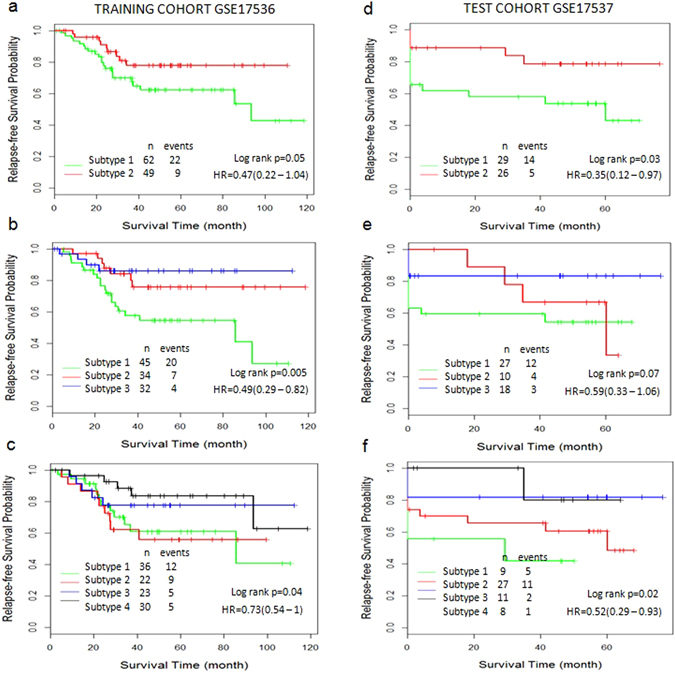
The molecular subtypes are associated with clinical outcomes in CRC. (**a**–**c**) Kaplan–Meier curve of relapse-free survival in training dataset GSE17536. The number of subtypes is from two to four respectively. (**d**–**f**) Kaplan–Meier curve of relapse-free survival in independent test dataset GSE17537. The number of subtypes is from two to four respectively. Hazard ratio (HR) was derived with 95% confidence interval.

To test the general applicability of the CSISCN, we applied it to CRC gene expression datasets. A CRC gene expression dataset GSE17536 with 111 samples was used as training cohort to develop the CSISCN for identifying molecular subtypes (Fig. 6a–c). Using optimized parameter σ derived from training cohort, the CSISCN was then evaluated using 55 samples in an independent dataset GSE17537 (Fig. 6d–f). In this analysis, the parameter σ = 20 was identified with the smallest p-value in CSISCN from CRC training cohort GSE17536. Figure 6a showed that the subtype 1 group had significantly worse relapse-free survival than the subtype 2 group. It illustrated that the patients were separated into three subtypes with significantly different relapse-free survival (Fig. 6b). We observed that the patients were stratified into four subtypes with significantly different relapse-free survival (Fig. 6c). Still, Fig. 6d showed that the subtype 1 group had significantly worse relapse-free survival than the subtype 2 group. We noticed that the patients were stratified into four subtypes with significantly different relapse-free survival (Fig. 6f).

**Figure 7:**
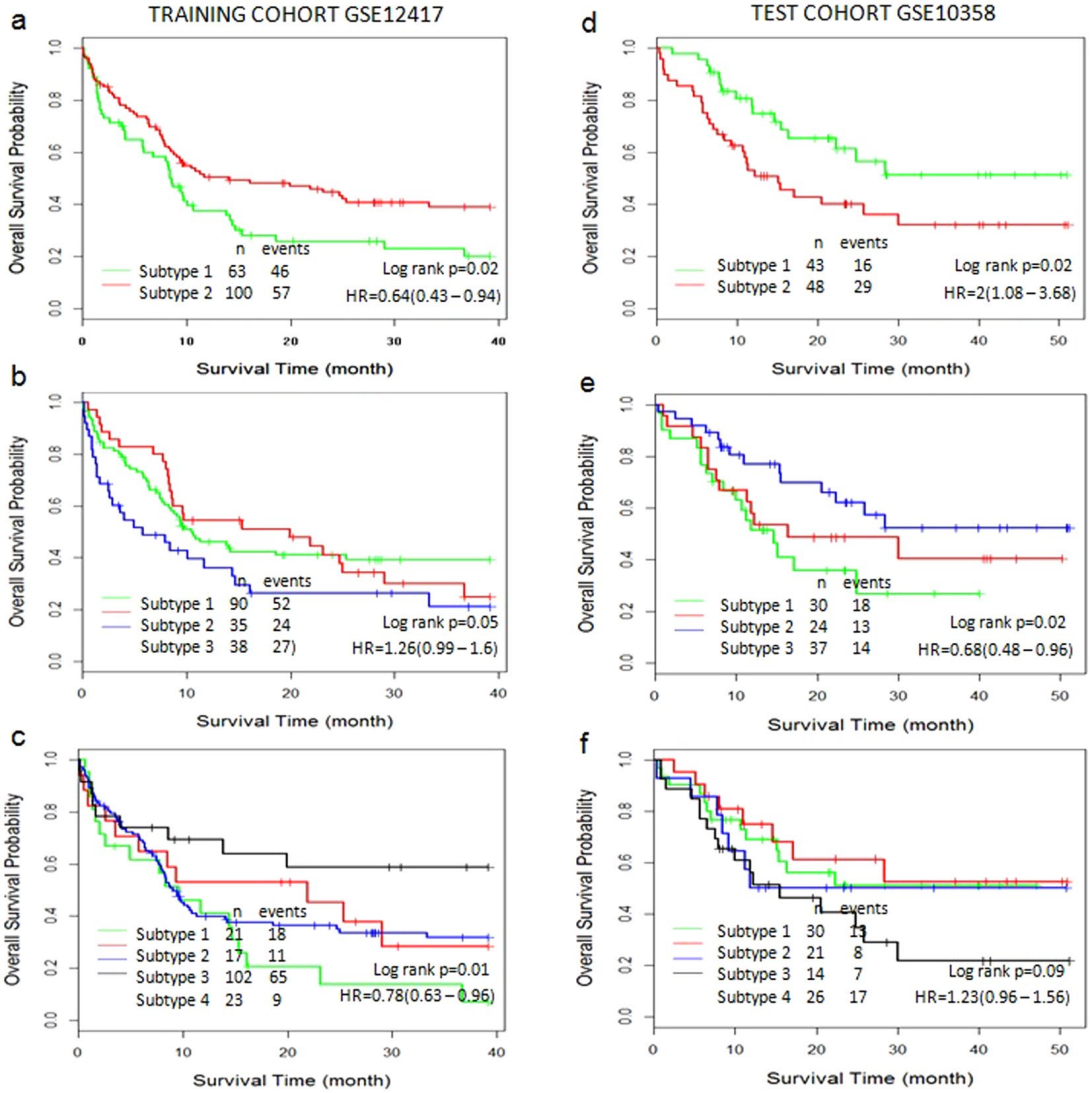
The Kaplan–Meier plot of patients stratified by the molecular subtypes in AML. (**a**–**c**) Kaplan–Meier curve of overall survival for two, three and four subtypes in training dataset GSE12417. (**d**–**f**) Kaplan–Meier curve of overall survival for two, three and four subtypes in independent test dataset GSE10358. Hazard ratio (HR) was derived with 95% confidence interval.

In addition, CSISCN was applied for AML gene expression datasets to further validate the general adaptability. Similar to the above analysis, we collected gene expression dataset GSE12417 (Fig. 7a–c) as training cohort to develop the CSISCN and kept GSE10358 (Fig. 7d–f) as an independent test dataset. In this analysis, the parameter σ = 30 was identified with the smallest p-value in CSISCN from AML training cohort GSE12417. The subtype 1 group had significantly worse overall survival than the subtype 2 group (Fig. 7a). We observed that the patients were separated into three subtypes with significantly different overall survival (Fig. 7b) and four subtypes with significantly different overall survival (Fig. 7c) respectively. Still, the subtype 2 group had significantly worse overall survival than the subtype 1 group (Fig. 7d). Figure 7e also showed the patients were separated into three subtypes with significantly different overall survival. In summary, these results were consistent with the observations in breast cancer and further demonstrated that CSISCN could identify molecular subtypes with distinct clinical outcome.

### The CSISCN is valid for the different numbers of molecular subtypes

In order to evaluate the validity of the CSISCN for the number of molecular subtypes, we tested different numbers of molecular subtypes for each cancer type. According to the association between the molecular subtype and real prognosis in GSE25055, statistically significant differences were found in the stratified patients with log-rank p-values less than 0.05 (Figs 3a–c, 4a,b and 5a–c). Similar performances were obtained for molecular subtype based stratification of patients in the independent validation datasets GSE25065 (Figs 3d,e, 4d,f and 5e) and GSE6532 (Figs 3g–i, 4g,i and 5g,i) respectively. These results suggested that the CSISCN was reasonably effective for the different numbers of molecular subtypes.

As depicted in Fig. 6, the similar results were observed when the CSISCN was applied for CRC gene expression datasets GSE17536 and GSE17537 respectively. As shown in Fig. 7, it suggested that the molecular subtypes with distinct clinical outcomes of AML identified in the training set could be rediscovered in the validation dataset. These results were consistent with the observations in breast cancer and further demonstrated the validity of the CSISCN for different numbers of molecular subtypes.

### The CSISCN identifies molecular subtypes for improving clinical and molecular relevance

We compared the CSISCN approach with the state of the art unsupervised method consensus clustering approach. In this analysis, we performed the comparisons with different molecular subtypes of each cancer type. Table 2 illustrated the log-rank p-values of CSISCN and consensus clustering from the training cohorts and independent test datasets. The p-values less than 0.05 were regarded as statistical significance.

**Table 2:** Comparison of CSISCN, consensus clustering (CC) and spectral clustering (SC) approach for identifying molecular subtypes.

		GSE 25055	GSE 25065	GSE 6532	GSE 17536	GSE 17537	GSE 12417	GSE 10358
k = 2	CSISCN	**0**.**02**	**0**.**04**	0.03	0.05	**0**.**03**	**0**.**02**	0.02
	CC	0.39	0.13	0.13	**0**.**04**	0.32	0.55	**0**.**01**
	SC	0.04	0.05	**0**.**02**				
k = 3	CSISCN	0.04	**0**.**01**	**0**.**005**	**0**.**005**	**0**.**07**	0.05	**0**.**02**
	CC	**0**.**01**	0.08	0.81	0.96	0.16	**0**.**046**	0.03
	SC	0.03	0.11	0.06				
k = 4	CSISCN	**0**.**04**	0.11	**0**.**05**	**0**.**04**	**0**.**02**	**0**.**01**	0.09
	CC	0.20	0.64	0.85	0.25	0.52	0.36	**0**.**02**
	SC	0.09	**0**.**08**	0.12				
k = 5	CSISCN	0.04	**0**.**03**	**0**.**001**	**0**.**05**	**0**.**01**	**0**.**02**	0.08
	CC	0.21	0.14	0.03	0.09	0.28	**0**.**02**	**0**.**02**
	SC	**0**.**03**	0.05	0.04				
k = 6	CSISCN	**0**.**01**	0.09	**0**.**11**	**0**.**03**	**0**.**03**	0.12	**0**.**07**
	CC	0.05	**0**.**04**	0.23	0.15	0.05	**0**.**02**	0.09
	SC	0.05	0.09	**0**.**11**				
k = 7	CSISCN	**0**.**11**	**0**.**05**	**0**.**004**	**0**.**01**	**0**.**07**	**0**.**02**	0.05
	CC	0.28	0.24	0.03	0.09	0.33	0.05	**0**.**02**
	SC	**0**.**11**	0.15	0.09				
k = 8	CSISCN	**0**.**01**	0.28	**0**.**005**	**0**.**04**	**0**.**02**	**0**.**02**	0.19
	CC	0.02	**0**.**05**	0.05	0.08	0.13	0.06	**0**.**02**
	SC	0.17	0.18	0.26				
k = 9	CSISCN	**0**.**01**	**0**.**01**	0.11	**0**.**001**	**0**.**02**	**0**.**02**	0.15
	CC	0.05	0.17	**0**.**03**	0.15	0.04	0.12	**0**.**01**
	SC	0.09	0.35	0.28				
k = 10	CSISCN	**0**.**03**	**0**.**09**	**0**.**03**	**0**.**001**	0.04	**0**.**08**	**0**.**03**
	CC	0.17	0.54	0.04	0.75	**0**.**01**	0.29	0.24
	SC	0.14	0.51	0.13				

The log-rank p-values from Kaplan-Meier survival analysis are derived for the training and test cohort of different cancer types. k is the number of molecular subtypes. The bold number is the best result for each k subtype.

According to log-rank p-values of breast cancer GSE25055, CSISCN achieved better performance than consensus clustering approach (Table 2). Similar results were also derived for different molecular subtypes based differentiated patients in the independent validation datasets GSE25065 and GSE6532 respectively. For breast cancer GSE25065, CSISCN achieved the best clustering performance for three and nine molecular subtypes respectively. For breast cancer GSE6532, CSISCN achieved the lowest log-rank p-value of 0.001 for five molecular subtypes. Thus, it suggested that CSISCN achieved p-values which tended to be more statistically significant than consensus clustering.

For CRC cohort GSE17536, it was showed that CSISCN achieved better clustering performance than consensus clustering approach with different molecular subtypes (except for k = 2). Meanwhile, CSISCN achieved p-values for different molecular subtypes (except for k = 3, 6) which are more statistically significant than consensus clustering in AML cohort GSE12417. Compared with consensus clustering, CSISCN achieved better clustering performance for the identification of different molecular subtypes in the independent test datasets CRC cohort GSE17537 and AML cohort GSE10358 respectively. Indeed, these results reproduced the outcomes in breast cancer and further proved the progress in the CSISCN for identifying molecular subtypes.

### The CSISCN improved clustering performance compared with spectral clustering method

To further validate the effectiveness of the CSISCN, we compared it with standard spectral clustering method for further analysis. A Matlab implementation available of spectral clustering was used to identify the molecular subtypes from breast cancer GEPs. The Gaussian similarity function was used for spectral clustering to construct the similarity matrix. The parameter **σ** was set among the candidate set {**20**, **30**, **40**, **50**}, evaluated with log-rank p-value over 10 runs and then identified with the smallest p-value. We tested different number of molecular subtypes for comparison. As shown in Table 2, CSISCN outperformed spectral clustering significantly for breast cancer GSE25055 (k = 2, 4, 6, 8, 9, 10), GSE25065 (k = 2, 3, 5, 7, 9, 10) and GSE6532 (k = 3, 4, 5, 7, 8, 9, 10) respectively. The results thus suggested that CSISCN achieved better clustering performance compared with spectral clustering.

We compared CSISCN with spectral clustering method in terms of running time. We performed the runtime experiments on a computer with 3.2 GHz CPUs and 16 GB of memory, without exploiting multi-core parallelization. In the implementation of CSISCN, the running time was separated into three sections including the calculation of similarity matrix, eigendecomposition and k-means implementation respectively. The total runtime for different molecular subtypes with CSISCN was reported in Table 3. The results suggested that CSISCN achieved a faster computational speed than spectral clustering method (Table 3).

**Table 3:** Runtime for the breast cancer gene expression datasets with CSISCN and standard spectral clustering (SC) method.

Subtype	Method	GSE25055	GSE25065	GSE6532
k = 2	CSISCN	538s	473s	514s
	SC	685s	617s	643s
k = 3	CSISCN	551s	479s	522s
	SC	699s	625s	657s
k = 4	CSISCN	560s	484s	531s
	SC	703s	627s	670s

## Discussion

The identification of molecular subtype is critical to the development of therapeutic strategy and the understanding of significant heterogeneity for cancer patients. In this analysis, our hypothesis is that spectral clustering method could identify molecular subtypes in correlation with survival outcomes. Furthermore, we developed the accurate subtype identification method for identifying molecular subtypes and thus improving clinical and molecular relevance. The CSISCN was then applied on different types of cancer to identify molecular subtypes and demonstrated superior performance as compared to consensus clustering and spectral clustering methods.

In our analysis, we used quantile normalization across the experiments to make comparable distributions for all samples. However, strong batch effect remained after this processing step. Importantly, further application of a gene-wise z-score transformation for each dataset separately effectively reduced the batch effect. Considering unsupervised clustering method is able to summarize and explain key features corresponding to several classes to which the data belong, we apply spectral clustering using Nyström approximation for the discovery of molecular subtypes. This unsupervised clustering method is then designed to capture the underlying cluster structures for a lower-dimensional representation of the data^28, 42^. Specially, this clustering method discards the structures which are always dominated by the arbitrariness of the sample noise and characterized by over-fitting in unsupervised learning^28, 42^. The results thus demonstrated that CSISCN was able to achieve significantly better performance for three cancer types. As compared to consensus clustering, CSISCN used the pairwise similarities of samples and smaller subset of dense similarity matrix, which thus achieved significantly better performance for the identification of molecular subtypes. Indeed, spectral clustering using Nyström approximation samples columns of the affinity matrix and approximates the full matrix by using correlations between the sampled columns and the remaining columns^24^, which is different from general spectral clustering method. Importantly, sampling-based spectral decomposition technique, Nyström method, provides a powerful alternative for approximate spectral decomposition. They often operate on a small part of the original matrix and eliminate the need for storing the full matrix^43^. While the general spectral clustering method needs to construct an adjacency matrix and calculate the eigen-decomposition of the corresponding Laplacian matrix, the Nyström approximation method is typically used for efficiently computing an approximate solution of the eigen-problem. Spectral clustering is mainly based on the manifold assumption, and this assumption is not applicable to identifying a low-dimensional data manifold of high-dimensional data. Actually, the clustering performance of SC will be degraded and even become worse than K-means clustering when high-dimensional data do not display a low-dimensional manifold structure clearly^44^. In this analysis, spectral clustering using Nyström approximation has been applied to discover the underlying cluster structure which is a lower-dimensional representation of high-dimensional gene expression data and thus identifies the molecular subtypes of cancer. In our study, we noticed the difference between performance gain for various k clusters when CSISCN is compared with two general clustering methods. It is interesting to see that the performance gain is very large for nine and ten clusters in GSE25055 and GSE25065 respectively (Table 2), and the results suggests the CSISCN shows great potential for large k clusters. Moreover, we also repeated the parameter selection for ten times when possible to obtain a more robust estimation. In the implementation of spectral clustering using Nyström approximation, a closer look of the results found that the performance could be very similar (or equal) when we run the algorithm ten times for the identical parameter value.

However, our findings come up with some caveats. Our analysis is restricted by the availability of genomic data for cancer patients. Moreover, we also notice some exceptional performance between CSISCN and consensus clustering in log-rank p-values (Table 2). Specifically, CSISCN performed the clustering performance with different log-rank p-values between training dataset and test dataset for each cancer type. One possible explanation is the biological difference that we observe the reality between different patient cohorts. For example, in the AML study, the training dataset GSE12417 was from a US population while the test dataset GSE10358 was from an European population. Another possible explanation is that the different class proportions between the training and the test datasets could result in the biases for clustering performance. For example, in the breast cancer study, the proportion between non-recurrence and recurrence patients is 3.7:1 in GSE25055 and 1.9:1 in GSE6532 respectively. Interestingly, this problem is popular in microarray studies with the small sample size.

With increasing available gene expression data from different types of cancer, CSISCN could bridge unsupervised learning method and accurate subtype discovering tool for the identification of cancer molecular subtypes. In summary, CSISCN shows the great potential for the discovery of molecular subtypes for human cancers.
